# Multipath Routing in Wireless Sensor Networks: Survey and Research Challenges

**DOI:** 10.3390/s120100650

**Published:** 2012-01-09

**Authors:** Marjan Radi, Behnam Dezfouli, Kamalrulnizam Abu Bakar, Malrey Lee

**Affiliations:** 1 Faculty of Computer Science and Information Systems, Universiti Teknologi Malaysia (UTM), Johor 81310, Malaysia; E-Mails: radi@ieee.org (M.R.); dezfouli@ieee.org (B.D.); knizam@utm.my (K.A.B.); 2 Center for Advanced Image and Information Technology, School of Electronics & Information Engineering, ChonBuk National University, 664-14, 1 Ga, DeokJin-Dong, Jeonju, ChonBuk 561-756, Korea

**Keywords:** wireless sensor networks, multipath routing, concurrent multipath routing, alternative path routing, load distribution, energy efficiency, reliability, QoS

## Abstract

A wireless sensor network is a large collection of sensor nodes with limited power supply and constrained computational capability. Due to the restricted communication range and high density of sensor nodes, packet forwarding in sensor networks is usually performed through multi-hop data transmission. Therefore, routing in wireless sensor networks has been considered an important field of research over the past decade. Nowadays, multipath routing approach is widely used in wireless sensor networks to improve network performance through efficient utilization of available network resources. Accordingly, the main aim of this survey is to present the concept of the multipath routing approach and its fundamental challenges, as well as the basic motivations for utilizing this technique in wireless sensor networks. In addition, we present a comprehensive taxonomy on the existing multipath routing protocols, which are especially designed for wireless sensor networks. We highlight the primary motivation behind the development of each protocol category and explain the operation of different protocols in detail, with emphasis on their advantages and disadvantages. Furthermore, this paper compares and summarizes the state-of-the-art multipath routing techniques from the network application point of view. Finally, we identify open issues for further research in the development of multipath routing protocols for wireless sensor networks.

## 1. Introduction

Recent advances in wireless communication technologies and the manufacture of inexpensive wireless devices have led to the introduction of low-power wireless sensor networks. Due to their ease of deployment and the multi-functionality of the sensor nodes, wireless sensor networks have been utilized for a variety of applications such as healthcare, target tracking, and environment monitoring [1]. The main responsibility of the sensor nodes in each application is to sense the target area and transmit their collected information to the sink node for further operations. Resource limitations of the sensor nodes and unreliability of low-power wireless links [2], in combination with various performance demands of different applications impose many challenges in designing efficient communication protocols for wireless sensor networks [3]. Meanwhile, designing suitable routing protocols to fulfill different performance demands of various applications is considered as an important issue in wireless sensor networking. In this context, researchers have proposed numerous routing protocols to improve performance demands of different applications through the network layer of wireless sensor networks protocol stack [4,5]. Most of the existing routing protocols in wireless sensor networks are designed based on the single-path routing strategy without considering the effects of various traffic load intensities. In this approach, each source node selects a single path which can satisfy performance requirements of the intended application for transmitting its traffic towards the sink node. Although route discovery through single-path routing approach can be performed with minimum computational complexity and resource utilization, the limited capacity of a single path highly reduces the achievable network throughput [6,7]. Furthermore, the low flexibility of this approach against node or link failures may significantly reduce the network performance in critical situations. For instance, whenever the active path fails to transmit data packets (as a result of limited power supply of the sensor nodes, high dynamics of wireless links and physical damages), finding an alternative path to continue data transmission process may cause extra overhead and delay in data delivery. Therefore, due to the resource constraints of sensor nodes and the unreliability of wireless links, single-path routing approaches cannot be considered effective techniques to meet the performance demands of various applications. In order to cope with the limitations of single-path routing techniques, another type of routing strategy, which is called the multipath routing approach has become as a promising technique in wireless sensor and *ad hoc* networks. Dense deployment of the sensor nodes enables a multipath routing approach to construct several paths from individual sensor nodes towards the destination [8]. Discovered paths can be utilized concurrently to provide adequate network resources in intensive traffic conditions. Alternatively, each source node can use only one path for data transmission and switch to another path upon node or link failures. The latter one is mainly used for fault-tolerance purposes, and this is known as *alternative path routing*.

In the past decade, multipath routing approach has been widely utilized for different network management purposes such as improving data transmission reliability, providing fault-tolerant routing, congestion control and Quality of Service (QoS) support in traditional wired and wireless networks. However, the unique features of wireless sensor networks (e.g., constrained power supply, limited computational capability, and low-memory capacity) and the characteristics of short-range radio communications (e.g., fading and interference [9,10]) introduce new challenges that should be addressed in the design of multipath routing protocols. Accordingly, existing multipath routing protocols proposed for traditional wireless networks (such as *ad hoc* networks) cannot be used directly in low-power sensor networks. During the past years, this issue has motivated the research community of wireless sensor networks to develop multipath routing protocols which are suitable for sensor networks.

There are several papers surveying proposed routing protocols for wireless sensor networks. These surveys describe and analyze the general routing strategies proposed for sensor networks [4,5]. However, none of these literatures has provided a comprehensive taxonomy on the existing multipath routing protocols for wireless sensor networks. Al-Karaki *et al.* [4] presented routing challenges and design issues in wireless sensor networks. They classified all the existing routing strategies based on the network structure and protocol operation. Alwan *et al*. [11] provided a brief overview on the existing fault-tolerant routing protocols in wireless sensor networks and categorized these protocols into retransmission-based and replication-based protocols. Tarique *et al.* [12] and Mueller *et al.* [13] classified the existing multipath routing protocols in *ad hoc* networks based on the primary criterion used in their design. Accordingly, the principal motivation of conducting this research was lack of a comprehensive survey on the proposed multipath routing protocols for wireless sensor networks. To the best of our knowledge, this paper is the first effort to classify and investigate the operation as well as benefits and drawbacks of the existing multipath routing protocols in sensor networks.

The rest of this paper is organized as follows. Section 2 provides an overview on the main challenges in designing routing protocols for wireless sensor networks and presents a brief classification on the existing protocols in these networks. Primary motivations behind using multipath routing approach in wireless sensor networks and key issues in designing multipath routing protocols are discussed in Section 3. Section 4 introduces a comprehensive taxonomy of the existing multipath routing protocols in wireless sensor networks. This classification provides a deep analysis on the most recently proposed multipath routing protocols, highlighting their advantages and disadvantages. Section 5 examines the main features of different classes of multipath routing protocols and their suitability for various applications. Finally, Section 6 concludes and identifies some of the future directions for further research in this area.

## 2. Routing in Wireless Sensor Networks

Since, data transmission from the target area towards the sink node is the main task of wireless sensor networks, the utilized method to forward data packets between each pair of source-sink nodes is an important issue that should be addressed in developing these networks.

Due to the intrinsic features of low-power wireless sensor networks, routing in these networks is much more challenging compared to the traditional wireless networks such as *ad hoc* networks [4,5]. First of all, according to the high density of sensor nodes, routing protocols should be able to support data transmission over long distances, regardless of the network size. In addition, some of the active nodes may fail during network operation due to energy depletion of the sensor nodes, hardware breakdowns or environmental factors, but this issue should not interrupt the normal network operation. Moreover, as sensor nodes are tightly limited in terms of power supply, processing capability, memory capacity and available bandwidth, routing and data dissemination should be performed with efficient network resource utilization. Furthermore, since the performance demands of the wireless sensor networks are application specific, routing protocols should be able to satisfy the QoS demands of the application for which the network is being deployed. For example, challenges in designing routing protocols for time critical applications (e.g., target tracking and disaster management) are different from issues that should be considered in developing routing protocols for other applications such as habitat monitoring.

According to the aforementioned differences between wireless sensor networks and traditional wireless networks, numerous routing protocols were proposed over the past decade to address the routing challenges imposed by the new features of sensor networks. Al-Karaki *et al*. [4] classified the existing routing protocols in wireless sensor networks from two different perspectives: (1) network structure and (2) protocol operation. From the network structure point of view, routing algorithms are classified as flat, hierarchical and location-based routing protocols. Flat routing protocols are designed for networks with homogenous nodes, *i.e.*, all the network nodes have the same processing and data transmission capabilities while their packet forwarding role is also similar. Directed Diffusion [14], Sensor Protocols for Information via Negotiation (SPIN) [15], Rumor Routing [16], Minimum Cost Forwarding Algorithm (MCFA) [17], and Energy-Aware Routing (EAR) [18] can be included in this category. According to the simple structure of the flat network architecture, this group of routing protocols demonstrates several advantages such as the low overhead of topology maintenance and the ability of multipath discovery. Hierarchical routing protocols were originally proposed to improve network scalability and energy efficiency through node clustering. In this group of routing protocols, all the sensor nodes are grouped into clusters and one node with more resources in each cluster is assigned as the cluster head. Each cluster head is responsible for processing the received data packets from its cluster nodes, communicating with other cluster heads or the sink node, and coordinating the cluster nodes. In contrast, all the cluster members should sense the environment and forward their collected data towards the respective cluster head for further operations. Although this approach can provide higher network scalability, clustering operation and cluster head replacement (which is required to prevent fast energy depletion of the cluster heads) impose high signaling overhead to the network. Several routing protocols such as Threshold-Sensitive Energy-Efficient Sensor Network Protocol (TEEN) [19], Adaptive Periodic Threshold-Sensitive Energy-Efficient Sensor Network Protocol (APTEEN) [20], Low-Energy Adaptive Clustering Hierarchy (LEACH) [21], Power-Efficient Gathering in Sensor Information Systems (PEGASIS) [22] and Two Tire Data Dissemination (TTDD) [23] fall in this category. Routing protocols in the last group utilize the exact location of the sensor nodes to make routing decisions [24,25]. The geographic locations of sensor nodes can be obtained directly using Global Positioning System (GPS) devices or indirectly by exchanging some information regarding to the signal strengths received at each node. However, since localization support requires specific hardware components and imposes significant computational overhead to the sensor nodes, this approach cannot be easily used in resource-constrained wireless sensor networks. Geographic and Energy-Aware Routing (GEAR) [24] and Geographic Adaptive Fidelity (GAF) [26] can be referred as the geographic routing protocols.

From the protocol operation perspective, all the existing routing protocols in the aforementioned categories can be further classified into negotiation-based, query-based, QoS-based, multipath-based and coherent-based protocols. The key idea in the design of negotiation-based routing protocols is to provide energy-efficient communication by reducing data redundancy during data transmission. In these protocols (e.g., SPIN family of protocols [15]), each sensor node adds a high-level data description to its collected data and performs some negotiations with its neighboring nodes to eliminate duplicated data packets. In the query-based routing protocols, a sink node propagates a query message throughout the network regarding the desired sensing task. If a node senses any related information, it sends back its collected data towards the sink node through the reverse path. Directed Diffusion [14] and Rumor Routing [16] are two examples of the primary query-based routing protocols. The third group of routing protocols (*i.e.*, QoS-based routing protocols) is mainly designed to satisfy QoS demands of different applications (e.g., delay, reliability, and bandwidth). The main aim of these approaches is to establish a trade-off between energy consumption and data quality. Sequence Assignment Routing (SAR) protocol [27], SPEED [28], Multipath Multi-SPEED (MMSPEED) [29], Energy-aware QoS Routing Protocol [30] and Delay-minimum Energy-aware Routing Protocol (DERP) [31] can be considered as the QoS-aware routing protocols. In contrast with single-path routing techniques, multipath routing protocols allow each source node to find multiple paths towards the sink node to improve network performance. There are several protocols in this category such as Braided Multipath Routing [32] and N-to-1 Multipath Routing Protocol [33]. Since, in wireless sensor networks, all the network nodes cooperatively process the flooded data in the network, the last group of routing algorithms is dedicated to the coherent data processing-based routing protocols. In this group, data packets are sent to the aggregators in order to reduce data redundancy. Therefore, energy efficiency is the main purpose of these routing protocols. Routing protocols such as Directed Diffusion [14], SPIN [15] and SAR [27] utilize data aggregation and can be categorized as the coherent data processing-based routing protocols. From this point onward, we focus on the multipath routing approach and the related issues that should be considered in the design of these protocols for wireless sensor networks.

## 3. Multipath Routing in Wireless Sensor Networks

Due to the limited capacity of a multi-hop path [6] and the high dynamics of wireless links [2,9], single-path routing approach is unable to provide efficient high data rate transmission in wireless sensor networks. Nowadays, the multipath routing approach is broadly utilized as one of the possible solutions to cope with this limitation. This section presents the primary motivations behind using multipath routing approach in wireless sensor networks. We also discuss the main design issues in the development of the existing multipath routing protocols.

### 3.1. Motivations for Using Multipath Routing Approach in Wireless Sensor Networks

As stated before, multipath routing technique has demonstrated its efficiency to improve wireless sensor and *ad hoc* networks’ performance. In the following, we review the performance gains that can be achieved through using multipath routing approaches.

*Reliability and Fault-Tolerance*: Due to the time-varying characteristics of low-power wireless links, dynamic network topology, and wireless interference, reliable data transmission in wireless networks is a challenging task [34,35]. The original idea behind using multipath routing approach in wireless sensor networks was to provide path resilience (against node or link failures) and reliable data transmission. In the fault tolerance domain, whenever a sensor node cannot forward its data packets towards the sink, it can benefit from the availability of alternative paths to salvage its data packets from node or link failures. Through this mechanism, as long as an alternative path is available from a target area towards the sink node, data forwarding can be continued without any interruption even in the case of path failure.

Multiple paths also can be used simultaneously to elevate data transmission reliability. There are two different approaches to provide reliable data transmission through concurrent multipath routing. The first approach is based on transmitting multiple copies of an original data packet over different paths to ensure packet recovery from several path failures. Through this technique, data transmission reliability can be guaranteed, if data forwarding over at last one path is done successfully. Erasure coding is another technique used by some of the existing protocols to provide desired reliability demand of different applications. Based on the utilized coding technique, each source node adds some additional information to the original data packets and then distributes the generated data packets over different paths. To reconstruct original packets, at last a certain number of transmitted data packets from each source node should be received by the sink node. Accordingly, if a few numbers of paths failed to deliver some data packets to the sink node, still the reliability of data transmission can be guaranteed through reconstructing data packets from successfully received data packets by the sink node.

*Load Balancing and Bandwidth Aggregation*: According to the resource limitations of wireless sensor nodes, intensive traffic loads in high-data rate applications are prone to congestion, which highly influences the network performance [36,37]. To handle this problem, data dissemination algorithms can profit from the high density of wireless sensor networks to increase network capacity by employing more network resources. For this purpose, multipath routing approaches can provide the best solution to support the bandwidth requirements of different applications and reduce the probability of network congestion through splitting network traffic over several paths [38–40]. Furthermore, distributing network traffic over more sensor nodes can result in even energy consumption among the sensor nodes and prolong the network lifetime. However, the broadcast nature of radio communications impedes achieving these goals. In fact, since in a single-channel wireless network, sensor nodes use a shared wireless channel to communicate with each other, concurrent utilization of adjacent paths results in intensive inter-path interference, which increases the probability of packet collision at the nodes along the active paths [41,42]. In the literature, this problem is called the *route coupling effect* [43] and specifically limits the performance of multipath routing protocols [44,45]. This issue imposes a big challenge in designing efficient multipath routing protocols. In order to reduce the effects of route coupling problem, *location-aware routing* is one of the most evident techniques to construct non-interfering paths [25,46]. Alternatively, other techniques such as directed antenna [47] and multi-channel data transmission [48–51] have been proposed to reduce inter-path interference. However, as location-aware routing protocols demand localization support, they impose significant overhead in terms of communication and computational complexity. Utilizing directed antennas for interference reduction also requires specific hardware equipment that may not be cost-effective in low-cost wireless sensor nodes. In addition, while multi-channel communication can increase network throughput and reduce inter-node interference, it requires a specific MAC layer mechanism that supports channel switching. More importantly, multichannel communication in the frequency band 2.4 GHz is highly affected by the external interference caused by 802.11 networks and Bluetooth devices. Accordingly, these approaches may not be efficient solutions to reduce the negative effects of interference in many applications. Wu *et al.* [52] introduced an indicator metric, which is called *correlation factor*, to measure the relative degree of inter-path independency. The correlation factor of two paths is defined as the number of links connecting two paths to each other. Since this approach requires the network connectivity graph to select the minimum interfering paths, using this technique in dense wireless sensor networks imposes high computational overhead.

*QoS Improvement*: QoS support in terms of network throughput, end-to-end latency and data delivery ratio is an important objective in designing multipath routing protocols for different types of networks [8,13]. Discovered paths with various characteristics can be utilized to distribute network traffic based on the QoS demands of the application for which the multipath routing protocol has been designed. For instance, time critical data packets can be transmitted through higher capacity paths with minimum delay while delay insensitive non-critical data packets can be forwarded through non-optimal paths with higher end-to-end delay. Furthermore, in contrast with the single-path routing techniques multipath routing approaches can preserve QoS demands of the intended application in the case of path failures through directing network traffic to the another active path. Still, due to the link layer issues in single-channel wireless networks, improving network throughput and data delivery ratio through concurrent multipath routing in sensor networks may not be as easy as wired networks.

### 3.2. Basic Principals in Designing Multipath Routing Protocols

Although the multipath routing approach has been employed for different purposes, the achieved performance gain is highly affected by the ability of the proposed protocol to construct an adequate number of high-quality paths. Each multipath routing protocol includes several components to construct multiple paths and distribute network traffic over the discovered paths. In the following, we describe these components in detail.

**Figure 1.**
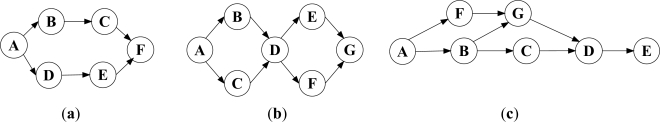
Various types of path disjointedness. (**a**) Node-Disjoint Paths; (**b**) Link-Disjoint Paths; (**c**) Partially Disjoint Paths.

*Path Discovery*: Since data transmission in wireless sensor networks is commonly performed through multi-hop data forwarding techniques, the main task of the route discovery process is to determine a set of intermediate nodes that should be selected to construct several paths from the source nodes towards the sink node. Different parameters are used in the existing multipath routing protocols to make routing decisions. Among these parameters, the amount of path disjointedness is the main criterion which is utilized by all the existing multipath routing protocols to discover several paths from each sensor node towards the sink node [8,12]. As depicted in Figure 1, discovered paths can be generally categorized as node-disjoint, link-disjoint, or partially disjoint paths. For node-disjoint paths, there is no common node or link among the discovered paths. Therefore, any node or link failure in a set of node-disjoint paths only affects the path, which contains the failed node or link. Since this kind of path disjointedness provides higher aggregated network resources, node-disjoint paths are preferred over link-disjoint and partially disjoint paths. Still due to the random deployment of the sensor nodes, it is difficult to discover a large set of node-disjoint paths between sensor nodes and sink node. In contrast, link-disjoint paths may contain several common nodes while there is no shared link between the paths. Accordingly, any node failure in a set of link-disjoint paths may deactivate several paths that share the failed node. Finally, partially disjoint paths can include multiple paths, which may share several links or nodes between different paths. In comparison with the aforementioned types of path disjointedness, any link or node failure in a set of partially disjoint paths may affect several paths. However, constructing multiple partially disjoint paths can be easily performed. Regarding to the advantages and disadvantages of different types of path disjointedness, network density and performance requirements of underlying application play an important role to make the best decision between using node-disjoint, link-disjoint or partially disjoint paths.

The amount of path disjointedness is the basic criteria that should be considered for discovering a set of paths, but due to the time-varying properties of radio communications and resource limitations of the sensor nodes, only considering this criterion may not result in the construction of high-capacity paths [2,53,54]. In some situations, merely assuming the amount of path-disjointedness for route discovery may lead to the construction of several low-quality paths. To address this problem, in addition to the amount of path disjointedness, different routing algorithms utilize various routing cost functions to make the best routing decision based on the application related performance demands. The main purpose of a routing cost function is to capture various properties of wireless links and sensor nodes to calculate cost of data transmission over different paths. To this aim, the employed routing cost functions in the existing multipath routing protocols are composed from several components to measure the capability of different nodes or links to provide performance demands of various applications (e.g., maximizing path throughput, minimizing end-to-end delay, improving network lifetime, and even traffic distribution). Path length, packet loss rate, delay and residual battery level of the sensor nodes are among the basic components of the routing cost functions utilized by the existing multipath routing protocols.

*Path Selection and Traffic Distribution*: After construction of multiple paths, another important issue that should be addressed is the selection of an adequate number of paths for data transmission purposes. According to the main intention of designing each multipath routing protocol, a certain number of paths should be selected to meet the performance demands of the intended application. Therefore, proposing a perfect path selection mechanism to choose a sufficient number of paths is the most important part of designing a high-performance multipath routing protocol. One protocol may decide to use only the best path for data transmission and keep additional paths as the backup paths for fault-tolerance purposes [12,32,55]. In contrast, another multipath routing protocol may utilize several paths concurrently to provide reliable data transmission or even traffic distribution [56–60]. Still, the number of selected paths plays an important role to improve different performance parameters. In fact, due to the wireless interference between nearby nodes, using all the constructed paths in single-channel wireless networks does not necessarily provide higher data transmission capacity. Nevertheless, data transmission through a few numbers of paths may not efficiently utilize the underlying resources of dense wireless sensor networks.

Once a set of paths are selected among the discovered paths, the multipath routing protocol should determine how to distribute network traffic over the selected paths. Based on the primary motivation behind the design of different multipath routing protocols they may utilize various traffic allocation mechanisms. For instance, transmission reliability can be guaranteed by introducing a certain degree of data redundancy in the data delivery process based on the reliability requirement of the underlying application. After that, the source node will utilize several paths to forward generated network traffic towards the sink node [57]. If the key idea is to improve performance demands such as throughput, data delivery ratio, delay, and lifetime, an efficient load distribution mechanism can be employed to distribute the overall network traffic over the constructed paths [41,45]. Furthermore, to improve resource utilization over individual paths, the injected traffic rate over each path should be calculated according to the path capacity.

*Path Maintenance*: Due to the resource constrains of sensor nodes and high dynamics of low-power wireless links, paths are highly error prone. Therefore, path reconstruction should be provided to reduce performance degradation. This is the main task of the path maintenance phase in multipath routing protocols. Path rediscovery process can be initiated in three different situations: (1) when an active path has failed, (2) when all the active paths have failed or, (3) when a certain number of active paths has failed. Since, the frequency of initiating route rediscovery process in the first approach is higher than for the two other approaches, using this strategy imposes a high overhead. Nevertheless, performing a route rediscovery process after the failure of all the active paths may significantly reduce network performance. Therefore, it seems that the third approach may represent a trade-off between the advantages and disadvantages of the first two approaches.

## 4. Taxonomy of the Existing Multipath Routing Protocols

Based on the discussed issues in Section 3.2, different applications demand for specific path selection and traffic distribution mechanisms.

**Figure 2.**
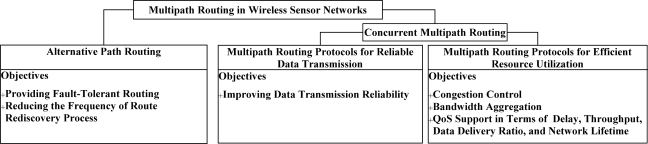
Taxonomy of the existing multipath routing protocols in wireless sensor networks.

Figure 2 introduces a comprehensive classification of the multipath routing protocols proposed for wireless sensor networks. The suggested taxonomy classifies the existing multipath routing protocols into three main categories (*i.e.*, alternative path routing, multipath routing for reliable data transmission, and multipath routing for efficient resource utilization), based on the employed path selection and traffic distribution mechanisms.

**Figure 3.**
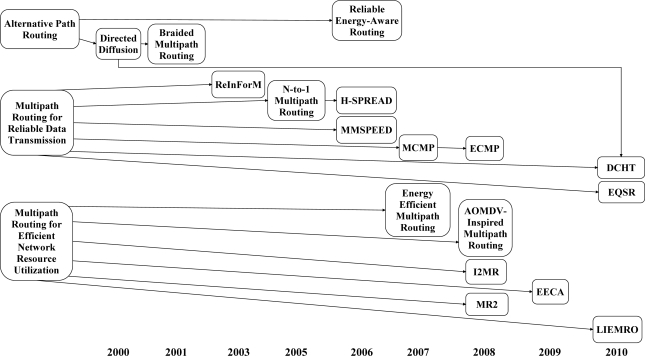
Taxonomy of the existing multipath routing protocols based on their historic design.

Figure 3 shows the three main classes of multipath routing approaches and their improvements over time.

### 4.1. Alternative Path Routing

Most of the existing multipath routing protocols in this class were mainly developed to provide fault tolerance at the network layer of protocol stack. Since providing fault tolerance was the primary motivation of utilizing multipath routing approaches for reliable data transmission over unreliable links, most of the early works on multipath routing technique fall in this category. As link and node failures are the main causes of path failures, the primary objective of these protocols is to guarantee certain performance parameters through preserving multiple alternative paths as the backup routes. This section introduces the ongoing research on the fault-tolerant routing using multipath routing approach. Table 1 summarizes few of the protocols from this category.

**Figure 4.**
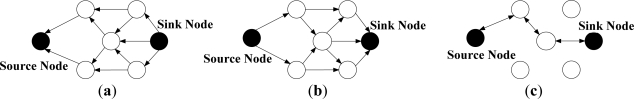
A sample scenario for path creation in Directed Diffusion. (**a**) Interest propagation; (**b**) Gradient setup; (**c**) Path reinforcement and data transmission

***Directed Diffusion*** [14] is a query-based routing protocol that uses the concept of multipath routing to provide path failure protection. Figure 4 shows the main operations of this protocol. According to Figure 4(a), routing operation is initialized by the sink node through flooding *interest* messages throughout the network. These *interest* messages contain some information regarding to the task that should be performed by the sensor nodes. During this stage, all the intermediate nodes cache the *interest* messages received from their neighbors for later use. Moreover, upon reception of an *interest* message, the receiver node creates a gradient towards the node from which this message has been received. As it can be seen from Figure 4(b), in this stage several paths can be discovered between each pair of source-sink nodes. After that, whenever a source node detects an event matched with the existing information in its interest table, it forwards its data packets towards the sink node through all the constructed gradients. The sink node receives its requested data through several paths with a low-data rate. Based on the packet reception performance over each path, the sink node can select the best path, *i.e.*, the path with minimum latency, for data transmission. For this, the sink node reinforces the selected path by sending low-rate *reinforcement* messages towards the source node. Then, the source node merely transmits its data towards the sink node through the selected path. This process is demonstrated in Figure 4(c). Furthermore, sink node continues to send low-rate *interest* messages over the remaining paths. This is to preserve the freshness of the established interest tables at the intermediate nodes, while it also maintains the discovered paths. When the active path fails to forward data packets, another available path can be used to provide fault-tolerant routing. Accordingly, whenever the data reception rate from the active path is reduced, the sink node reinforces the second available best path.

Simulation results [14] show that Directed Diffusion can preserve event delivery ratio in the case of any node or link failure along the active path. Moreover, this protocol reduces data transmission delays caused by path failure by decreasing the frequency of route rediscovery. As Directed Diffusion depends on low-rate flooding for route discovery and path maintenance, this protocol does not provide an efficient route discovery mechanism. Furthermore, according to the main operation of this protocol, it can only be used in query-driven applications.

**Figure 5.**
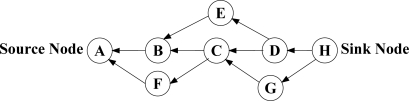
Braided multiple paths.

***Braided Multipath Routing Protocol*** [32] is a seminal multipath routing protocol proposed to provide fault-tolerant routing in wireless sensor networks. This protocol uses a similar approach as Directed Diffusion to construct several partially disjoint paths. A general form of the established paths is presented in Figure 5.

This protocol utilizes two types of path *reinforcement* messages to construct partially disjoint paths. Path construction is initiated through the sending of a *primary path reinforcement* message by the sink node to its best neighboring node towards the source node. For example, in Figure 5, the sink node sends the *primary path reinforcement* message to node *D*. When an intermediate node receives a *primary path reinforcement* message, it forwards this message to its best next-hop neighboring node towards the source node. This process is repeated until the *primary path reinforcement* message reaches the source node. In addition to the primary path construction process, source node and all the intermediate nodes along the primary path construct an alternative path around their next-hop neighboring nodes. This alternative path passes through the neighboring node, which is not included in the primary path. To this aim, whenever the sink and intermediate nodes send out the *primary path reinforcement* message, they also generate an *alternative path reinforcement* message and send this message to their next preferred neighboring node towards the source node. For instance, in Figure 5, the sink node sends an *alternative path reinforcement* message to node *G* in order to establish a backup path around node *D*. During this process, whenever an intermediate node, which is not a member of the primary path, receives an *alternative path reinforcement* message, it should forward this message to its best next-hop neighboring node. This process terminates upon reception of this message by one of the nodes along the primary path. As a result, each intermediate node along the primary path constructs a backup path around its next-hop neighboring node on the primary path via transmitting an *alternative path reinforcement* message. Through establishing a set of partially disjoint paths between the source and sink nodes, whenever the primary path fails to forward data packets towards the sink node, one of the constructed alternative paths can be utilized to avoid data transmission failure.

Simulation results [32] show the lower overhead of braided multipath routing approach compared to the idealized node-disjoint multipath protocol. Furthermore, through performance evaluation of the proposed protocol under a wide range of failure probabilities, it is demonstrated that the proposed approach provides about 50% higher resilience against path failures, compared to the idealized node-disjoint multipath protocol. However, since this protocol utilizes only one path for data transmission, the end-to-end throughput is limited to the capacity of a single path. Besides, since this approach is designed based on the principles of Directed Diffusion, the drawbacks of Directed Diffusion can be also applied to this protocol.

***Reliable and Energy-Aware Multipath Routing*** [55] is designed to mitigate the energy efficiency requirement of wireless sensor networks, while provides reliable data transmission through maintaining a backup path from each source node towards the sink node. Similar to the above presented protocols, the routing operation in this protocol is also initialized by the sink node. In this way, whenever the sink node receives an *interest* message from a source node and there is no active path towards the source node, it initiates a *service-path* discovery process through flooding a *service-path request* message. Upon reception of the *service-path request* message at the corresponding source node, the receiver node transmits a *service-path reservation* message towards the sink node (through the reverse path) to confirm the discovered path. While the *service-path reservation* message moves from the source node towards the sink node, whenever a node along the reverse path receives this message, it reserves a part of its residual battery level for data transmission over this path. The *service-path* construction process finishes by receiving the *service-path reservation* message at the sink node. Afterwards, the source node can transmit its data packets towards the sink node through the constructed path. After constructing the *service-path*, sink node initiates another path discovery process to establish a backup path towards the same source node by flooding a *backup path discovery* message. During this process, the intermediate nodes, which are not a member of the discovered *service-path*, broadcast the received *backup path discovery* message to their neighbors. Therefore, a node-disjoint path is created to provide fault tolerance in the case of *service-path* failure.

Although this protocol provides energy-efficient and reliable data transmission, however it suffers from the main disadvantage of the alternative path routing strategy: the end-to-end capacity is limited to the capacity of a single path. More importantly, this protocol neglects the effects of wireless interference and link unreliability on the required energy for successful data transmission.

### 4.2. Concurrent Multipath Routing

Thus far, the first group of multipath routing protocols, which are designed to fulfill the primary motivation of utilizing multipath routing, *i.e.*, fault-tolerant routing, were discussed. Ongoing research on multipath routing approach tries to cope with the resource limitations of low-cost sensor nodes through concurrent data forwarding over multiple paths. This section introduces some of the recent research in this area.

#### 4.2.1. Multipath Routing Protocols for Reliable Data Transmission

As it was pointed out in Section 3.1, in addition to the alternative routing techniques, concurrent multipath routing can be used to support reliable communication over unreliable low-power wireless links through introducing data redundancy during the data transmission process.

According to the operation of the protocols presented in the previous section, those protocols are designed to reduce the frequency of path rediscovery while they provide high path resilience against route failure. However, introduced protocols in this section utilize the packet replication technique or erasure coding in conjunction with the concurrent multipath routing technique to satisfy the reliability requirements of various applications. The rest of this section provides an in-detail explanation of the employed route discovery mechanisms in a few of the existing reliable-based multipath routing protocols and investigates about their advantages and disadvantages. Table 2 summarizes the main operation of the selected reliable-based multipath routing protocols to provide a fast overview over the main features of these protocols.

***Reliable Information Forwarding (ReInForm) Using Multiple Paths in Sensor Networks*** [57] uses the packet duplication technique to provide desired data transmission reliability for each application. In this approach, whenever a source node wants to forward its traffic towards the sink node, it first determines the required data transmission reliability based on the importance of the collected data. After that, the source node adds some information (e.g., local channel error rate, its hop count towards the sink node, and desired reliability) as Dynamic Packet State (DPS) fields to the data packets and sends multiple copies of the generated data packets over several paths. The source node determines the required number of paths to fulfill the reliability demands of the collected information according to the DPS fields of the data packets. During data transmission, all the intermediate nodes use the provided information by the DPS fields in the received data packets to determine the number of copies that should be transmitted to their next-hop neighboring nodes. This process continues until all the transmitted data packets reach to the sink node.

According to the main operation of this protocol, ReInForm tries to improve data transmission reliability through utilizing the packet duplication technique at all the involved sensor nodes in the data transmission process. Accordingly, the elevated reliability of this protocol is achieved at the high cost of energy consumption and bandwidth utilization, which is in contrast with the primary demands of resource-constrained sensor nodes.

**Figure 6.**
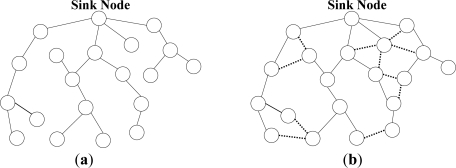
(**a**) Spanning tree constructed by initial flooding in N-to-1 Multipath Routing Protocol. (**b**) Multipath discovery using multipath extension flooding mechanism.

***N-to-1 Multipath Routing Protocol*** [33] is proposed according to the convergecast traffic pattern of wireless sensor networks. The main aim is to simultaneously discover multiple node-disjoint paths from all the sensor nodes towards a single sink node. Furthermore, during data transmission phase, all the intermediate nodes utilize a packet salvaging technique at each hop to improve data transmission reliability. The entire routing operation in N-to-1 multipath routing protocol is performed through a simple flooding strategy in two stages. The sink node starts the first stage of the route discovery process through broadcasting a *route update* message. This stage, which is called *branch-aware flooding*, utilizes the main benefit of a simple flooding technique to construct a spanning tree and discover several paths from sensor nodes towards a single sink node. During this phase, each sensor node that receives a *route update* message for the first time, selects the sender of this message as its parent towards the sink node. In addition, if an intermediate node overhears a *route update* message from another neighboring node that introduces an alternative node-disjoint path through a different branch of the spanning tree, it adds this path to its routing table. This process continues until all the sensor nodes discover their primary path towards the sink node and a spanning tree same as Figure 6(a) is constructed through all the nodes. After that, the second stage of this protocol is initialized in order to discover more paths from each sensor node towards the sink node with the use of *multipath extension flooding* technique. As it can be seen from Figure 6(b), each link between two individual nodes that belong to different branches of the constructed spanning tree can help to establish an additional path from these nodes towards the sink node. Accordingly, the main purpose of employing *multipath extension flooding* technique in the second stage is to exchange some information regarding to the discovered node-disjoint paths in the first stage between the nodes belong to different branches of the constructed spanning tree. At the end of this stage, a routing tree similar to the Figure 6(b) is constructed by all the sensor nodes. Finally, source nodes split their traffic into several segments and distribute these data segments over the discovered paths. In fact, this protocol utilizes the single-path forwarding strategy for transmitting each data segment, while all the intermediate nodes use an adaptive per-hop packet salvaging technique to provide fast data recovery from node or link failures along the active paths.

N-to-1 multipath routing protocol uses the broadcast nature of radio communications to construct several node-disjoint paths from sensor nodes towards the sink node without using additional control packets. This protocol also profits from the availability of several paths at the intermediate nodes to improve reliability of packet delivery by employing a per-hop packet salvaging strategy. Nevertheless, using such a simple flooding strategy cannot result in constructing high-quality paths with minimum interference. According to the operation of this protocol, all the constructed paths are located in physical proximity of each other and concurrent data transmission over these paths may reduce the network performance.

***H-SPREAD*** [61] combines the introduced path construction process in N-to-1 Multipath Routing Protocol with a hybrid data transmission technique to improve reliability and security of data transmission in wireless sensor networks. H-SPEAD takes advantages of a *threshold secret sharing* scheme and path diversity of multipath data forwarding to increase path resilient against node failure or compromised paths. According to the security property of the *threshold secret sharing* scheme, data packets can be safely forwarded towards the sink node even when a small number of nodes or paths have failed or are compromised during the data transmission process. In this algorithm, the source node divides each data packet to the multiple shares, *M_1_*, *M_2_*, *M_3_*,…, *M_n_*, through using the secret sharing strategy and then transmits them towards the sink node through different paths. Based on the special characteristics of the *threshold secret sharing* mechanism, even when a certain number of paths have failed due to link or node failures, the original message can still be retrieved via other received shares at the destination node. However, since this approach utilizes the N-to-1 multipath routing algorithm to construct multiple paths, this protocol may suffer from the effects of wireless interference. Therefore, high packet loss ratio caused by interference can reduce the probability of successful packet retrieval at the sink node. Moreover, H-SPREAD only improves reliability and security of data delivery in the network, but it cannot enhance security of individual nodes.

**Figure 7.**
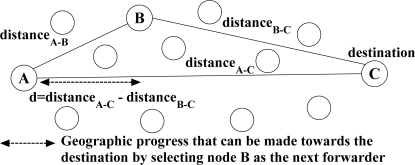
Progress speed from node A to node B towards the destination node.

***Multipath Multispeed Protocol (MMSPEED)*** [29] is designed based on the cross-layer design approach between network and MAC layer to provide QoS differentiation in terms of reliability and timeliness. From a timeliness perspective, MMSPEED extends the SPEED protocol [28] through introducing multiple speed levels to guarantee timeliness packet delivery. The utilized speed notion in this protocol can be realized through Figure 7. Suppose node *A* forwards a data packet to its immediate neighboring node *B,* which can reduce the remaining geographic distance to the destination (*i.e.*, node *C*) about *d* meters. According to the estimated delay of data transmission over link *A–B* (*i.e.*, *delay_A−B_*), the achievable progress speed towards the destination through forwarding this data packet to the node *B* can be calculated as *Speed_A–B_* = (*distance_A–C_* − *distance_B–C_*)/*delay_A–B_*. In the reliability domain, reliability demands of different applications are satisfied through using a probabilistic multipath forwarding strategy.

In order to satisfy the delay requirements of various applications, MMSPEED extends the SPEED protocol to provide different speed layers over a single network. Accordingly, for *M* virtual speed layers there exists *M* different *SetSpeeds*. In this protocol, data packets are assigned to the appropriate speed layer to be placed in the suitable queue according to their speed category. After that, data packets are serviced in the FCFS policy. This mechanism ensures that high-priority packets are serviced before low-priority packets. However, as contention-based MAC protocols utilize CSMA/CA mechanism to perform channel access [62,63], employing a local priority data transmission scheme at the network layer does not necessarily prioritize data transmission at the link layer. Therefore, MMSPEEAD benefits from a prioritized medium access mechanism through cross-layer interactions.

According to the above descriptions, whenever a source node wants to forward a data packet towards the destination, it determines the speed requirement of the data packet based on its distance to the destination and its specified end-to-end deadline. Then, the classifier of the source node selects the corresponding speed layer that can meet the speed requirements of the data packet. The selected speed layer module performs all the subsequent routing decisions for data packet forwarding during the data transmission process. These routing decisions are made based on the amount of speed progress that can be achieved by each intermediate node. Furthermore, if an intermediate node receives a data packet and it perceives that this packet cannot meet its specified deadline through the selected speed layer, the receiver node can set another speed layer to satisfy the deadline requirement of the packet. From reliability perspective, MMSPEED benefits from path diversity property of multipath routing approach to guarantee reliability requirements of each data packet. This protocol provides reliability differentiation through controlling number of active paths and sending multiple copies of the original data packets over several paths. Accordingly, each intermediate node selects a set of next-hop neighboring nodes towards the destination node based on the estimated packet loss rate over each link and their geographic distance from itself.

As mentioned above, MMSPEED provides a probabilistic QoS guarantee in two different domains through combining geographic forwarding technique with a multipath routing approach. To satisfy different delay requirements, each intermediate node tries to forward its received data packet to the neighboring node, which is closer to the destination node in order to provide a good speed progress. However, according to the experimental results provided in [9], probability of successful data transmission over low-power wireless links highly depends on the sender-receiver distance and interference power of the receiver. Therefore, using geographic routing with greedy forwarding does not necessarily improve network performance metrics. Moreover, since data transmission over long links exacerbate the required energy for data transmission, this protocol cannot support long-life applications.

***Multi-Constrained QoS Multipath Routing (MCMP)*** [60] is mainly designed to provide soft-QoS guarantee in terms of reliability and delay. The end-to-end soft-QoS problem is formulated as a probabilistic programming problem and then it is converted into a deterministic linear programming using an approximation technique. Therefore, MCMP is developed according to the linear programming approach which is a deterministic approximate of the defined end-to-end soft-QoS problem. Using Equations (1) and (2), MCMP maps the delay and reliability of the links along different paths towards the sink node to the end-to-end delay and reliability demands of various applications:

(1) $$L_{i}^{d}=\frac{D-D_{i}}{h_{i}}$$

(2) $$L_{i}^{r}{=}^{h_{i}}\sqrt{R_{i}}$$

where $L_{i}^{d}$ and $L_{i}^{r}$ represent the delay and reliability requirements at node *i*. *D_i_* is the delay experienced by a packet at node *i. R_i_* is the fraction of the reliability requirement assigned to the path passing through node *i*, and *h_i_* is the hop count from node *i* to the sink node.

**Figure 8.**
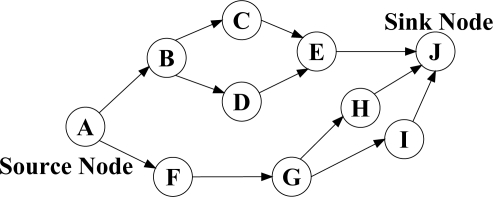
Partially disjoint paths established by MCMP.

MCMP utilizes two different strategies to satisfy delay and reliability demands of wireless sensor network applications. During the route discovery process, all the intermediate nodes utilize Equation (1) to choose the neighboring node that fulfills the delay requirement of the intended application. To satisfy reliability, each node selects one or a set of its neighboring nodes, which additively provides the desired reliability towards the sink node. Therefore, at the end of the route discovery process, each source node has discovered a set of partially disjoint paths that can additively satisfy delay and reliability demands of the target application. Figure 8 demonstrates a set of discovered paths using MCMP protocol. According to the structure of the constructed paths, source and intermediate nodes, which have discovered multiple sub-paths towards the sink node, should send several copies of the original data packets to the sink node through different sub-paths to provide reliability. For instance, in Figure 8, node *G* should forward two copies of its received data packets towards the sink node through node *H* and node *I*.

The introduced data redundancy of MCMP is the main disadvantage of this protocol. Furthermore, since partially disjoint paths are usually located nearby, high data rate transmission causes significant interference. This highly affects the maximum achievable data transmission rate using this protocol.

**Figure 9.**
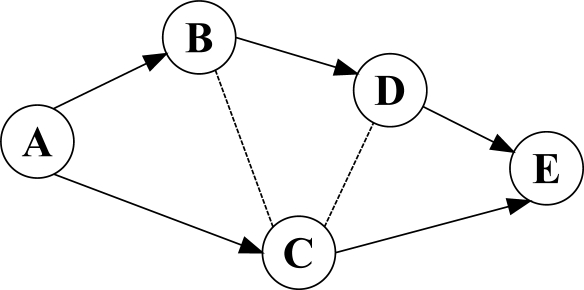
Link selection according to the geo-spatial energy consumption constraint.

***Energy Constrained Multipath Routing (ECMP)*** [64] is the extended version of MCMP to provide energy-efficient communication, while it also satisfies the delay and reliability requirements of each application. In the MCMP protocol described earlier, intermediate nodes select the set of their neighboring nodes that satisfies the delay and reliability requirements of the data source, regardless of the energy consumed for data transmission over individual links. In contrast, ECMP introduces an energy optimization problem. This problem is constrained by delay, reliability and geo-spatial energy consumption to provide multi-constrained QoS routing in sensor networks. Accordingly, the main motivation in designing ECMP is to support multi-constrained QoS routing with minimum energy consumption. To demonstrate this issue, consider Figure 9, where node *A* has two neighboring nodes that can equivalently satisfy the delay and reliability requirements of the intended application. As it can be seen from this figure, the distance between node A and node B is shorter than the distance between node *A* and node *C*. Since the required energy for data transmission can be related to the distance between sender and receiver [65], energy consumption for data transmission over link *A-B* is lower than the energy consumption for data transmission over link *A–C*. Therefore, selecting node *B* as the next-hop neighboring node *A* can result in lower energy consumption. However, in MCMP, nodes randomly select their next-hop neighboring nodes without considering the amount of energy consumption over the chosen link. Therefore, compared to MCMP, ECMP refines the set of next-hop nodes to a smaller set through considering the energy efficiency of the links towards the neighboring nodes.

According to the experimental results [64], both MCMP and ECMP provide an equivalent delivery ratio and transmission delay. However, ECMP results in lower energy consumption, compared with MCMP.

***Delay-Constrained High-Throughput Protocol for Multipath Transmission (DCHT)*** [56] is the modified version of Directed Diffusion [14] that propounds the idea of using multipath routing approach to support high-quality video streaming in low-power wireless sensor networks. DCHT introduces a novel path reinforcement method and uses a new routing cost function, which considers the expected transmission count (ETX) [2] and delay metrics to discover high-quality paths with minimum end-to-end latency. Similar to the Directed Diffusion, routing operation in this protocol is initialized by flooding an *interest* message throughout the network. Moreover, in order to calculate data transmission latency over each path, sink node adds a timestamp (*t_0_*) to the *interest* message. When a source node can provide the data requested by the sink node, it broadcasts *explore data* packets towards the sink node through the established gradients in the first stage. Upon reception of an *explore data* packet at an intermediate node, it uses Equations (3) and (4) to calculate the cost of data transmission over the sub-path from which this packet is received:

(3) $$\mathrm{Path\_Cost}={\mathrm{Path\_ETX}}^{\alpha }\times {\mathrm{Path\_Delay}}^{\beta }$$

(4) $$\mathrm{Path\_ETX}={\text{Max}}_{i=0}^{N-3}\left(\sum_{j=i}^{i+2}{\mathrm{ETX}}_{j}\right)$$

After that, the receiver node only broadcasts the lowest calculated cost to its next-hop neighboring nodes. In Equation (4), *N* indicates the number of traversed hops and *ETX_j_* identifies the ETX value of *j*th hop. In order to address the route coupling effect, ETX value of each link is estimated according to the experienced Signal-to-Noise Ratio (SNR) over that link.

When the sink node receives an *explore data* packet that cannot meet the delay requirement of the intended application, or whenever the path discovery timer expires, the sink node starts to reinforce multiple node-disjoint paths for data transmission. To satisfy the delay constraint of the intended application, the sink node investigates the end-to-end delay of the *explore data* packets received through different paths and only reinforces the paths with acceptable end-to-end delays. The path reinforcement is performed through sending *reinforcement* messages over the selected paths towards the source node. Whenever a *reinforcement* message is received by an intermediate node, it searches its local candidate table and selects one of its best next-hop neighboring nodes that does not belong to any other path between the source and sink node. When the source node receives the transmitted *reinforcement* messages by the sink node, it divides its traffic to several streams using the *Multiple Description Coding (MDC)* algorithm. This algorithm tries to improve data transmission reliability through forwarding each generated stream over two different paths.

The utilized path reinforcement strategy and routing metric in this protocol greatly improves the performance of the original Directed Diffusion by constructing multiple high-quality low-delay paths. When the target source node floods an explore data packet to the network, each node estimates the ETX value of its neighboring nodes based on its experienced SNR. Then, in the path reinforcement process, the sink node uses the ETX value of the discovered paths to select minimum interfering paths. However, this technique cannot accurately calculate the exact value of the experienced interference during the data transmission process. The actual interference strength during the data transmission process highly depends on the traffic load of the interfering nodes. If the interfering nodes, which are located near an active path, do not involve in any data forwarding process, these nodes cannot impose any interference on the active path. Furthermore, each generated data stream should be transmitted through two different paths to provide a certain level of data transmission reliability. However, due to the random topology of the wireless sensor networks, constructing a sufficient number of node-disjoint paths to support high-rate multimedia streaming may not be feasible.

***Energy-Efficient and QoS-based Multipath Routing Protocol (EQSR)*** [66] is one of the recently proposed protocols designed to satisfy the reliability and delay requirements of real-time applications. EQSR improves reliability through using a lightweight XOR-based Forward Error Correction (FEC) mechanism, which introduces data redundancy in the data transmission process. Furthermore, in order to fulfill the delay requirements of various applications, this protocol utilizes a service differentiation technique through employing a queuing model to manage real-time and non-real-time traffic. EQSR initializes through broadcasting a *HELLO* message by all the sensor nodes. During this phase, sensor nodes collect information regarding to the cost of data transmission though their neighboring nodes. In the second phase of this protocol, the sink node starts the route discovery process by sending a *Route-request* message to its preferred neighbor selected by Equation (5). Intermediate nodes use Equation (5) to select the most preferred next-hop neighboring node towards the source node from their neighboring set *N*. This process continues among the intermediate nodes until the source node receives a *Route-request* message transmitted by the sink node:

(5) $$\mathrm{Next\_hop}={\mathrm{Max}}_{y\in N_{x}}\left\{{\alpha E}_{\mathrm{resd},y}+{\beta B}_{\mathrm{buffer},y}+{\gamma I}_{\mathrm{interference},\mathrm{xy}}\right\}$$

where *N_x_* is the neighbor set of node *x. E_resd,y_* and *B_buffer,y_* indicate the residual battery level and available buffer size at neighbor *y*, respectively. *I_interference,xy_* is the experienced SNR over the link between node *x* and node *y*. All the sensor nodes calculate the values of these parameters for their neighboring nodes during the first stage of this protocol.

Besides the primary-path establishment process, the sink node also starts to construct additional paths by sending subsequent *Route-request* messages to its next-preferred neighboring nodes. Whenever all the possible paths between a pair of source-sink nodes are discovered, a set of paths will be selected based on the probability of successful data transmission over each path. Furthermore, according to the propagation delay of the *Route-request* messages, EQSR estimates the data transmission delay of the paths and dedicates the best *L* paths for real-time traffic and the remaining paths for non-real-time traffic. At the last stage of this protocol, EQSR uses a lightweight XOR-based FEC algorithm to calculate Error Correction Codes (ECC) for data packets. Finally, the source node distributes its traffic over the selected paths according to their end-to-end delay.

While EQSR reduces transmission delay and improves reliability, nevertheless, the FEC mechanism which is used to compute ECCs and retrieval of the original messages, imposes high control overhead. Furthermore, like DCHT, this protocol uses a flooding strategy to estimate the experienced SNR over wireless links at the initialization phase and uses these values to discover the minimum interfering paths. However, the employed routing cost function cannot lead to the construction of interference-minimized paths. In fact, using a simple flooding strategy during the neighbor discovery phase may exaggerate the exact value of mutual interference between different paths.

#### 4.2.2. Multipath Routing Protocols for Efficient Network Resource Utilization

With respect to the limitations of tiny sensor nodes, the key idea behind the development of this protocol category is to balance network traffic and resource utilization throughout the network. This section is dedicated to describe some of the most recently proposed protocols in this group of multipath routing protocols. Table 3 provides an in-depth comparison of the presented routing protocols based on the details of their employed route discovery and traffic distribution algorithms.

***Energy-Efficient Multipath Routing Protocol*** [67] exploits the path diversity provided by multipath routing approach to prolong network lifetime by distributing network traffic over multiple node-disjoint paths. When an event occurs in the network, a sensor node in the event area is selected as the source node and initiates the route discovery process. Accordingly, the selected source node transmits multiple *Route-request* messages to its neighboring nodes. These *Route-request* messages include different path *IDs* to construct multiple node-disjoint paths from the selected source node towards the sink node. During the route discovery process, all the intermediate nodes select one of their best next-hop neighboring nodes towards the sink node, which satisfy Equation (6) and it is not included in any other path:

(6) $$\mathrm{Next\_hop}={\text{arg min}}_{b\in N_{\alpha }}\left\{{\left(1-\frac{e_{b,\mathrm{residual}}}{e_{b,\mathrm{init}}}\right)}^{\left[\frac{\beta (1-(\Delta d+1))}{d_{\mathrm{ay}}}\right]}\right\}$$

where *N_a_* represents the neighboring set of node *a. d_ay_* is the distance (in hop count) between node *a* and sink node, *d_by_* is the distance (in hop count) between node *b* and sink node, and Δ*d* is the difference between *d_ay_* and *d_by_*. *e_b,residual_* and *e_b,init_* represent the residual and initial battery level of node *b*, respectively.

Upon reception of the first *Route-request* message by the sink node, it sets a timer to fulfill the path establishment process in an acceptable period. Therefore, all the paths discovered after the timer timeouts are considered as low-quality paths and the sink node discards the *Route-request* messages received from these paths. Then, the sink node assigns different data rates to the established paths using Equation (7). Sink node uses the *ASSIGN* messages to inform the selected source node about the assigned data rate of each path. Source node starts data transmission upon the reception of the *ASSIGN* messages:

(7) $$r_{j}=\frac{R}{P_{j}}\sum_{i=1}^{N}P_{i},j=1,2,\ldots ,N$$

Assuming *N* paths between a pair of source-sink nodes, *r_j_* is the assigned data rate to the *j*th path, *R* is the requested data rate (by the application) that should be arrived at the sink node. *p_i_* and *p_j_* are the costs of *i*th and *j*th paths.

The main advantage of this protocol is to prolong network lifetime by distributing network traffic over several paths according to the cost of data transmission over these paths. The residual battery level of the sensor nodes and their distance to the sink node are considered as the main parameters in the route discovery and load distribution algorithms. However, the interference level experienced by the intermediate nodes (along nearby paths) and its effects on the network performance is disregarded. On the other hand, as demonstrated in [59,68], a lower number of interference-minimized paths provides higher performance compared to the situation in which more number of paths is established without considering the effects of interference. Nevertheless, this protocol establishes and utilizes all the discovered node-disjoint paths.

**Figure 10.**
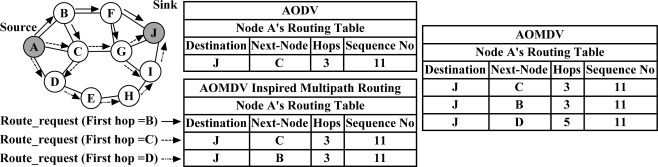
Constructed routing tables at the source node by AODV, AOMDV and AOMDV-Inspired Multipath routing protocols.

***AOMDV-Inspired Multipath Routing Protocol*** [69] is designed based on the multipath version of AODV (*i.e.*, AOMDV [70]) to achieve energy-efficient and low-latency communication in wireless sensor networks through using cross-layer information. Path construction is similar to the mechanism introduced in AOMDV with a few improvements. While AOMDV tries to discover all the possible link-disjoint paths between each pair of source-sink nodes, the AOMDV-Inspired Multipath Routing Protocol uses different routing table management strategy to construct only hop count optimal paths towards the destination node. With this protocol, the sink node confirms an additional path only if its first hop is different from the previously discovered paths and if this path provides the same hop count towards the sink node. Otherwise, if the sink node receives a *Route-request* message with the lower hop count than the existing routes (established from the same source node), it substitutes all the previously established paths by the newly discovered path. Figure 10 demonstrates the routing tables constructed at the source node by AODV, AOMDV and AOMDV-Inspired Multipath Routing Protocol.

AOMDV does not introduce any load distribution mechanism to split network traffic over the established paths. AOMDV-Inspired Multipath Routing Protocol utilizes the information provided by the MAC layer to reduce data transmission latency. To this aim, during data transmission process, each intermediate node searches its routing table and forwards its received data packets to the next-hop neighboring node that wakes up earlier. While this MAC layer technique can reduce the transmission delay and interference, it requires all the sensor nodes to be aware about their neighboring nodes’ timing information. Furthermore, similar to the *ad hoc*-based routing protocols, this protocol should flood the whole path information throughout the network during the route discovery phase. This flooding process imposes significant overhead to the resource-limited sensor nodes.

**Figure 11.**
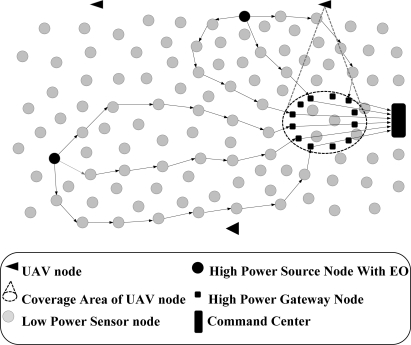
Assumed network structure in the design of I2MR. Constructed paths between each source node and command centre are demonstrated.

***Interference-Minimized Multipath Routing Protocol (I2MR)*** [45] aims to support high-rate streaming in low-power wireless sensor networks through considering the recent advances in designing high-bandwidth backbone networks. I2MR tries to construct zone-disjoint paths and distributes network traffic over the discovered paths by assuming a special network structure and the availability of particular hardware components. Figure 11 demonstrates a simple schematic of the network structure assumed in this protocol.

**Figure 12.**
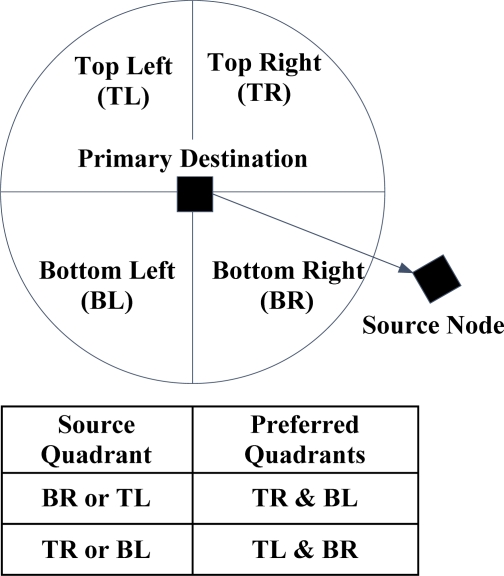
Preferred quadrants for the secondary and backup destinations based on the source node location.

All the deployed gateway nodes are assumed as the final destinations and it is supposed that these nodes are directly connected to the command center using non-interfering and high-capacity links. In I2MR, the source node utilizes two paths for data transmission and keeps only one backup path towards the central command center. The route discovery phase includes three main steps: in the first step, each source node selects one gateway node as its primary gateway node and constructs the shortest possible path towards this gateway node. Then, in the *interference-zone marking* step, one and two-hop neighboring nodes of all the intermediate nodes along the first path are marked as the interference zone of the primary path. Finally, in the last stage, the primary gateway node determines the preferred quadrants from which the secondary and backup gateway nodes should be selected. As it is shown in Figure 12, these quadrants are determined based on the location of the source node. Furthermore, the preferred gateway nodes should be located beyond the interference rang of the primary gateway node and they should have less distances to the source node than the other candidate gateway nodes. When the secondary and backup gateway nodes are determined, the source node starts to construct the secondary and backup paths through the nodes that are not marked as the interference-zone of the primary path. At the end of the path construction process, source node loads the primary and secondary paths with the highest possible data rate and preserves the third path to achieve prompt packet recovery upon path failure. During the data transmission process, whenever an intermediate node along an active path detects a long-term congestion, it should notify the source node to reduce its injected data rate.

The simulation results indicate the higher performance of I2MR compared to the standard AODV and a simple node-disjoint multipath routing protocol [45]. However, the achieved performance improvement requires a special network structure and particular hardware components, which may not be feasible for many applications. In addition, due to the high complexity of the introduced zone-marking algorithm, this mechanism cannot effectively construct interference-minimized paths. Moreover, source nodes construct the three shortest paths (*i.e.*, with minimum hop count) towards three separate gateway nodes to reduce the effects of wireless interference among the successive nodes along a path. However, due to the time-varying properties of low-power wireless links, data transmission over long hops results in increased packet loss ratio.

***Maximally Radio-Disjoint Multipath Routing (MR2)*** [71] utilizes an adaptive incremental technique to construct minimum-interfering paths, which satisfy the bandwidth requirements of multimedia applications. Additional paths are constructed whenever the active paths cannot provide the bandwidth requirements of the available network traffic. Like the other query-based routing protocols [14,55,56], the sink node initializes the route discovery process by flooding the network with a *request* message. Upon reception of the *request* message by the immediate neighboring nodes of the sink node, the receiver node adds its ID to the received *request* message as the path ID and rebroadcasts this message. Then, whenever a node receives a *request* message, it first checks the reported path ID and if it has not any path from the introduced source node (*i.e.*, path ID) towards the sink node, it should add the reported path to its routing table. Otherwise, if the included path ID in the received *request* message already exists in the routing table of the receiver, the introduced path should be replaced with the previous one if it provides a path with lower hop count. If the received *request* message causes an update operation on the routing table, the receiver node should rebroadcast the *request* message. This process is continued until the *request* message is received by a sensor node that can provide sink node with the requested data. At this time, source node starts packet transmission towards the sink node through the shortest discovered path. In order to address the mutual interference problem between adjacent paths, all the intermediate nodes along the active path should notify their neighboring nodes to act as the passive nodes in order to prevent them from participating in any route discovery process. Therefore, during the data transmission process, intermediate nodes that receive a data packet should send a *bepassive* message to all of their neighboring nodes except their next and previous-hop neighbors along the active path. Using this mechanism, whenever an additional path should be constructed (to provide sufficient bandwidth for data transmission), passive nodes are unable to respond to any *request* message.

This protocol eliminates the negative effects of wireless interference by putting some nodes in the passive state. Simulation results confirm that MR2 improves the overall data reception rate at the sink node more than 70% and 30% compared to a multipath routing approach without interference-awareness and a single-path routing scheme, respectively. Still, this protocol suffers from two main drawbacks: first, MR2 is only suitable for query-driven applications; second, the utilized flooding strategy for constructing non-interfering paths imposes a high control overhead.

**Figure 13.**
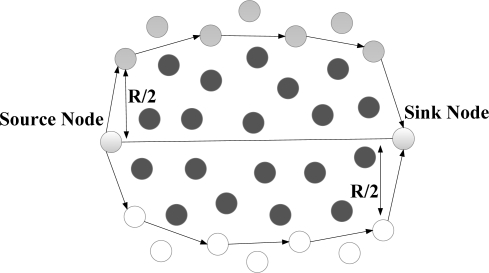
A simple example of the constructed paths by EECA.

***Energy-Efficient and Collision-Aware Multipath Routing Protocol (EECA)*** [25] is an on-demand multipath routing protocol and uses the location information of all the sensor nodes to establish two collision-free paths between a pair of source-sink nodes. EECA aims to reduce the negative effects of wireless interference through constructing two paths in both sides of the direct line between the source-destination pair. Furthermore, the distance between these two paths is more than the interference range of the sensor nodes. This is demonstrated in Figure 13. In the first stage of the route discovery process, the source node checks its neighboring nodes to find two distinct groups of the nodes on both sides of the direct line between the source-destination pair. Figure 13 represents these two groups with white and gray circles. After finding these neighboring sets, the source node broadcasts a *Route-request* packet towards these nodes to establish two node-disjoint paths. During the route discovery process, intermediate nodes utilize the same technique (used at the source node) to select their next-hop neighboring nodes and broadcast the received *Route-request* packet towards the sink node. Upon reception of a *Route-request* packet by an intermediate node, the receiver node uses a back-off timer to restrict the overhead introduced by the route discovery flooding. Before broadcasting the received *Route-request* packet by the intermediate nodes, they set a back-off timer according to their distance from the sink node and their residual battery level. Neighboring nodes with higher residual battery and shorter distance to the sink node select shorter back-off timer. Therefore, at each stage of the *Route-request* flooding only one node wins to broadcast its received *Route-request* packet towards the sink node. Upon reception of the *Route-request* packet at the sink node, it sends a *Route-reply* packet in the reverse path towards the source node. When the source node receives a *Route-reply* packet, it can transmit its traffic through the established path.

Although EECA tries to discover the two shortest paths such that their distance from each other is more than interference range of the sensor nodes, it needs the nodes to be GPS-assisted and relies on the information provided by the underlying localization update method. These requirements increase the cost of network deployment and intensify the communication overhead, specifically in large and dense wireless sensor networks. In addition, as low-power wireless links exhibit significant signal variations over time, calculating the interference range of the sensor nodes based on the distance may not result in an accurate interference estimation [72]. Moreover, while transmitting data over minimum-hop paths can theoretically reduce end-to-end delay and resource utilization, however, using such paths in low-power wireless networks increases the probability of packet loss and intensifies the overhead of packet retransmission over each hop.

***Low-Interference Energy-Efficient Multipath Routing Protocol (LIEMRO)*** [59,68] improves the performance demands of event-driven sensor networks (e.g., delay, data delivery ratio, throughput, and lifetime) through construction of an adequate number of interference-minimized paths. LIEMRO utilizes an adaptive iterative approach to construct a sufficient number of node-disjoint paths with minimum interference from each event area towards the sink node. Whenever an event occurs in the sensor field and there is no active path for data transmission towards the sink node, the selected source node starts to establish the first path by transmitting a *Route-request* message towards the sink node. During this stage, source node and all the intermediate nodes select one of their next-hop neighboring nodes using Equations (8) and (9):

(8) $${\mathrm{Next\_hop}}_{i}=\left\{j|\forall j\in N_{i}\;\mathrm{and}\;{\mathrm{Cost}}_{i,j}={\mathrm{Min}}_{j\in N_{i}}\left({\mathrm{Cost}}_{i,j}\right)\right\}$$

(9) $${\mathrm{Cost}}_{i,j}=\left({\mathrm{accETX}}_{i,\mathrm{sink}}\right)\cdot \left(\frac{1}{{\mathrm{resBatt}}_{j}}\right)\cdot \left(1+{\mathrm{interferenceLevel}}_{j}\right)$$

In Equation (8)*N_i_* represents the neighboring set of node *i*. In Equation (9), *resBatt_j_* is the residual battery level of node *j*, *interferenceLevel_j_* is the experienced interference level at node *j*, and *accETX_i,sink_* is the accumulated ETX value from node *i* to the sink node through neighboring node *j*. ETX value of a link is calculated as *1/pq*, where *p* and *q* indicate the probability of successful forward and backward packet reception over that link, respectively. During the network initialization and neighbor discovery phase, the accumulated ETX value of all the sensor nodes towards the sink node are calculated through constructing the optimal spanning tree using the ETX cost.

Upon reception of the first *Route-request* message by the sink node, it confirms the discovered path by forwarding a *Route_reply* message along the reverse path. While the *Route_reply* message moves from sink node towards the source node, whenever a node overhears this message it updates its interference level value based on the backward packet reception probability (*i.e.*, *q*) of the node from which this message has been overheard. When the source node receives a *Route_reply* packet, it transmits its data packets through the constructed path and starts the construction of another path by sending a new *Route-request* message towards the sink node. Path construction process continues in an iterative manner as long as the sink node realizes that using a new path results in higher end-to-end throughput; otherwise, if the last established path reduces the end-to-end throughput, sink node asks the source node to disable the last constructed path. Upon establishing a new path (*i.e.*, when the source node receives a *Route_reply* packet), the source node transmits a portion of its traffic through this path using a quality-based load distribution algorithm. The proposed load balancing algorithm calculates the optimal traffic rate of the established paths based on their accumulated residual battery level, experienced interference level, and the probability of successful forward and backward packet reception over the links of a path.

LIEMRO improves the performance demands of event-driven applications through distributing network traffic over high-quality paths with minimum interference. This protocol utilizes a dynamic path maintenance mechanism to monitor the quality of the active paths during network operation and regulates the injected traffic rate of the paths according to the latest perceived paths quality. Therefore, it accounts for the temporal variations of the low-power wireless links and adjusts traffic distribution accordingly. However, similar to the most of the previously discussed protocols, LIEMRO does not consider the effects of buffer capacity and service rate of the active nodes to estimate and adjust the traffic rate of the active paths.

## 5. Application Related Issues

Nowadays, multipath routing is widely considered as a promising approach to cope with the limitations of wireless sensor networks and it can be used to improve the performance demands of different applications. However, while a multipath routing approach improves the performance requirements of a specific application, it may negatively affect the performance requirements of another application. For example, as transmitting multiple copies of data packets increases delivery reliability, it also reduces network lifetime and capacity due to the imposed overhead. Therefore, choosing a right multipath routing approach is highly application dependent and involves the trade-off between several performance parameters. Table 4 summarizes the main motivation and employed approaches behind the development of the protocols presented in the previous section.

As mentioned earlier, the first motivation behind utilizing multipath routing approaches in wireless sensor networks was to improve path resilience against route failures through the alternative path routing technique. Since, the key idea in this approach is to use one path for data transmission and reserve the alternative paths as the backup paths in the case of route failures, these protocols suffer from the same main drawback of single-path routing approaches, *i.e.*, limited end-to-end capacity. The improvement of this approach over single-path routing is that this technique increases network performance (it reduces the consumed energy per bit, loss rate caused by path failure, and path reconstruction delay), while it also reduces the frequency of the route rediscovery process. As partially disjoint paths can provide fault-tolerant routing through the alternative path routing approach with minimum cost, most of the multipath routing protocols in this category use this kind of path disjointedness to reduce the imposed overhead by the route discovery and maintenance processes.

Some of the critical applications (e.g., battlefield surveillance and intrusion detection) require high data transmission reliability; accordingly, the second group of multipath routing protocols is mainly designed to cope with the time-varying properties and unreliability of low-power wireless links. As we described in Section 4.2.1, these protocols provide reliable communication through utilizing the path diversity nature of multipath routing approach and introduce data redundancy into the data delivery process (e.g., transmitting multiple copies of original packets, or erasure coding). Although the efficiency of these protocols in improving data transmission reliability is demonstrated through extensive performance evaluations [29,57,60,64], still, they suffer from the high overhead caused by transmitting multiple copies of data packets and utilizing coding scheme.

Due to the resource limitations of sensor nodes and low capacity of individual paths, recently, multipath routing approach is broadly utilized to increase network capacity under high traffic conditions (e.g., multimedia streaming [46,56,66,71]). As it is observable from Figure 3 and Table 4, most of the recently proposed multipath routing protocols utilize concurrent multipath routing to support even traffic distribution (to balance resource utilization) and provide the required bandwidth of high-rate applications. On the other hand, when data is transmitted through multiple nearby paths, the overall capacity of each path reduces due to the interference caused by the other paths. From the MAC layer point of view, a node belonging to a path may sense the carrier busy while a nearby node in an adjacent path is transmitting. In addition, an ongoing transmission on a path may be affected by the interference induced from nearby paths. These issues cause higher medium access delay, increased packet loss and elevated end-to-end latency of the packets being transmitted to the sink node. Therefore, the use of concurrent multipath routing cannot necessarily satisfy the performance demands of high data rate applications. While the protocol designer should consider the required end-to-end latency and capacity of the underlying applications to establish a tradeoff between the inter-path distances and the length of each path, special MAC layer mechanisms may be required to schedule per-hop transmissions based on the experienced interference. Specifically, as cross-layer interactions of the network and MAC layer can result in higher performance [62,63,73], multipath routing can be significantly improved through utilizing cross-layer principles.

## 6. Conclusions and Future Directions

This paper provides a comprehensive analysis of the most recently proposed multipath routing protocols for wireless sensor networks. Nowadays, multipath routing techniques are considered an efficient approach to improve network capacity and resource utilization under heavy traffic conditions. With respect to the recent advances in the development of multipath routing protocols for wireless sensor networks, there is a need to investigate the significance as well as the detailed operation and classification of the proposed approaches. To fill this gap, in this paper we have attempted to identify the challenges pertaining to the design of multipath routing protocols for wireless sensor networks. In addition, we have highlighted the main advantages of using multipath routing approach to satisfy the performance requirements of different applications. This paper also introduces a new taxonomy on the multipath routing protocols designed for wireless sensor networks. The provided classification is performed based on the employed path utilization methods that can be used by multipath routing protocols to achieve various performance benefits. Tables 1–3 demonstrate detailed operational characteristic of the existing multipath routing protocols related to the different categories. Finally, all the presented multipath routing protocols in this paper are summarized in Table 4 to provide a fast overview of the main motivations behind their design and the methods employed to achieve the desired goals.

Although in the past years multipath routing has been researched through numerous studies, nevertheless, there are several important research issues that should be further investigated. These possible areas can be summarized as follows: first, cross-layer principles can be used to improve multipath routing protocols. For instance, the channel access delay and per-hop latency perceived at the MAC layer can be used at the network layer to achieve more accurate path quality estimation and rate assignment. Second, when multiple paths are in use, packet reception order at the sink node may be different from the packet transmission order at the source node. This issue affects the performance of applications such as multimedia streaming and wastes network resources. Finally, the development of multi-constrained QoS multipath routing protocols to guarantee the QoS demands of different applications is an open area. To this aim, different multipath routing approaches (such as using backup paths, sending multiple copies of data packets, and concurrent path utilization) should be integrated efficiently.

## Figures and Tables

**Table 1.** Summary of the multipath routing protocols with alternative path routing approach.

**Features**	**Path Disjointedness**	**Route Maintenance**	**Traffic Distribution**	**Number of Paths**	**Path Chooser**	**Improved Performance Parameters**
**Protocols**						
**Directed Diffusion**	Partially disjoint	New route discovery when all the active paths have failed	Not applicable	Not limited	Sink node	▪Data transmission delay caused by path failure ▪Packet loss rate caused by path failure
**Braided Multipath Routing**	Partially disjoint	New route discovery when all the active paths have failed	Not applicable	Not limited	Sink node	▪Data transmission delay caused by path failure ▪Packet loss rate caused by path failure ▪Route discovery and path maintenance overhead
**Reliable and Energy-Aware Routing**	Node-disjoint	New route discovery when the primary path has failed	Not applicable	Two paths	▪Source node ▪intermediate nodes	▪Packet loss rate caused by path failure ▪Network lifetime

**Table 2.** Summary of the selected multipath routing protocols which are designed to provide reliable data transmission.

**Features**	**Path Disjointedness**	**Route Maintenance**	**Traffic Distribution**	**Employed Reliability Mechanism**	**Number of Paths**	**Path Chooser**	**Improved Performance Parameters**
**Protocols**							
**ReInForm**	Link-disjoint	Not mentioned	Multiple copies of each packet	Copying the original packets	Based on the desired reliability	▪Source node	▪Reliability
**N-to-1 Multipath Routing**	Node-disjoint	Not mentioned	Per-packet splitting	Packet salvaging	Not limited	▪Source node ▪Intermediate nodes	▪Reliability
**H-SPREAD**	Node-disjoint	Not mentioned	Per-packet splitting	Erasure coding	Not limited	▪Source node ▪Intermediate nodes	▪Reliability ▪Security
**MMSPEED**	Partially disjoint	Not mentioned	Multiple copies of each packet	Copying the original packets	Based on the desired reliability	▪Source node ▪Intermediate nodes	▪Reliability ▪Delay
**MCMP**	Partially disjoint	Not mentioned	Multiple copies of each packet	Copying the original packets	Based on the desired reliability	▪Intermediate nodes	▪Data delivery ratio ▪Delay
**ECMP**	Partially disjoint	Not mentioned	Multiple copies of each packet	Copying the original packets	Based on the desired reliability and energy consumption for data transmission over individual links	▪Intermediate nodes	▪Network lifetime ▪Data delivery ratio ▪Delay
**DCHT**	Node-disjoint	Not mentioned	Two copies of each packet over two paths	Erasure coding	Not limited	▪Source node ▪Intermediate nodes	▪Data delivery ratio ▪Delay
**EQSR**	Node-disjoint	Not mentioned	Per-packet splitting	Erasure coding	Based on the probability of successful data delivery over the active paths	▪Source node	▪Data delivery ratio ▪Delay

**Table 3.** Summary of the multipath routing protocols mainly designed to provide efficient resource utilization.

**Features**	**Path Disjointedness**	**Route Maintenance**	**Traffic Distribution**	**Number of Paths**	**Path Chooser**	**Interference Avoidance Technique**	**Improved Performance Parameters**
**Protocols**							
**Energy-Efficient Multipath Routing**	Node-disjoint	When two or less than two paths are active	Per-packet splitting	Not limited	▪Sink node ▪Intermediate nodes	None	▪Network lifetime ▪Delay
**AOMDV-Inspired Multipath Routing**	Link-disjoint	When all the paths have failed	Per-packet splitting	Not limited	▪Sink node	None	▪Network lifetime ▪Delay
**I2MR**	Node-disjoint	When first and second paths have failed	Per-packet splitting	Three paths	▪Sink node ▪Intermediate nodes	Through using the exact location of the source and destination nodes	▪Throughput
**MR2**	Node-disjoint	When a path has failed	Per-packet splitting	Based on the bandwidth requirements of the target application	▪Sink node ▪Intermediate nodes	Through using the broadcast nature of wireless channel	▪Network lifetime ▪Throughput ▪Data delivery ratio
**EECA**	Node-disjoint	Not mentioned	Per-packet splitting	Two paths	▪Intermediate nodes	Through using the exact location of sensor nodes	▪Network lifetime ▪Data delivery ratio
**LIEMRO**	Node-disjoint	When less than two paths are active	Per-packet splitting	Based on the end-to-end throughput of the active paths	▪Sink node ▪Intermediate nodes	Through using the broadcast nature of wireless channel	▪Network lifetime ▪Throughput ▪Delay ▪Data delivery ratio

**Table 4.** Summary of the presented multipath routing protocols.

**Path Utilization**	**Motivation**	**Approach**	**Protocols**
**Alternative Path Routing**	Fault-Tolerant Routing	Path Switching	Directed Diffusion, Braided Multipath Routing, Reliable and Energy-Aware Routing
**Concurrent Multipath Routing**	Reliable Data Transmission	Copying the Original Packets	ReInForm, MMSPEED, MCMP, ECMP
		Erasure Coding	H-SPREAD, DCHT, EQSR
		Packet Salvaging	N-to-1 Multipath Routing
	Efficient Network Resource Utilization	Load Balancing	Energy-Efficient Multipath Routing, AOMDV-Inspired Multipath Routing, I2MR, MR2, EECA, LIEMRO
